# Progressive relaxation exercises: association with dyspnea, anxiety, and oxygen saturation among patients with chronic obstructive pulmonary disease

**DOI:** 10.3389/fresc.2026.1806416

**Published:** 2026-04-09

**Authors:** Doaa Samir Elsayed, Salwa Samir Kamel, Reham Adel Ebada, Hossam Hassan Sayed

**Affiliations:** 1 Department of Medical Surgical Nursing, Faculty of Nursing, Ain Shams University, Cairo, Egypt; 2 Department of Adult Nursing, Faculty of Nursing, Egyptian Chinese University, Cairo, Egypt; 3 Department of Chest Faculty of Medicine, Ain Shams University, Cairo, Egypt

**Keywords:** anxiety, chronic obstructive pulmonary disease, dyspnea, oxygen saturation, patients, progressive relaxation exercise

## Abstract

**Background:**

Progressive relaxation exercises may benefit chronic obstructive pulmonary disease (COPD) patients by reducing muscle tension, calming the nervous system, and alleviating stress, potentially improving dyspnea and anxiety. By promoting controlled breathing, these exercises could enhance overall quality of life. This study evaluates the effects of progressive relaxation on dyspnea, anxiety, and oxygen saturation in individuals with COPD.

**Methods:**

A quasi-experimental single-group pretest-posttest design was used. A purposive sample of 60 patients with COPD at the chest department and outpatient clinic recruited from Ain Shams Hospital, Cairo. Patients' socio-demographic characteristics and clinical data, the Dyspnea 12 questionnaire, the Beck Anxiety Inventory, and the performance checklist for progressive relaxation exercises. For primary pre–post comparisons within the same group, paired-samples *t*-tests were used for normally distributed continuous outcomes. Analyses were conducted in SPSS version 27; a two-sided *p*-value <0.05 was considered significant.

**Results:**

The study revealed that 51.7%, 50% and 40.7% of the studied patients experienced moderate and mild dyspnea pre-exercise, post-exercise, and in follow-up, respectively. 55%, 50% and 43.3% had moderate anxiety pre-exercises, mild anxiety post-exercises, and follow-up up respectively. 83.3% of the studied patients demonstrated a satisfactory level of performance.

**Conclusion:**

that there were statistically significant associations among the total scores of the Dyspnea-12 (D-12), the Beck Anxiety Inventory (BAI), and the Progressive Relaxation Exercise (PRE) checklist after one month of implementing progressive relaxation exercises in patients with COPD. These findings support developing a simplified, illustrated, comprehensive booklet including information about COPD, lifestyle changes for coping with this disease, and its therapeutic regimen.

## Introduction

Chronic obstructive pulmonary disease (COPD) is a leading cause of morbidity and mortality worldwide. The World Health Organization estimates that more than 200 million people live with COPD globally (1). Chronic bronchitis is an inflammatory condition of the airways that clinically manifests as an augmented productive cough lasting at least three months. These airway changes can be accompanied by dyspnea, recurrent infections, and persistent productive cough.

The primary clinical manifestations of COPD include dyspnea and reduced activity tolerance, with decreased respiratory muscle function identified as a major contributor to dyspnea in COPD patients (2). Dyspnea significantly limits physical activity and contributes to disability and anxiety. Progressive relaxation exercises are practical, evidence-based techniques that nurses can integrate into COPD management as part of pulmonary rehabilitation, and patients can readily perform at home. Evidence indicates that relaxation exercises in COPD can improve oxygen saturation and help alleviate symptoms such as dyspnea (3).

Patients with COPD may experience hypoxemia; hence, monitoring peripheral oxygen saturation (SPO2) during pulmonary rehabilitation is recommended. To assess the precision of SPO2 readings from wearable devices in COPD patients, both at rest and after physical exercise (4).

Historically, COPD treatment has centered on pharmacological management and symptomatic relief; however, there has been a shift toward comprehensive rehabilitation programs. Non-pharmacological interventions commonly employed for COPD symptom management include breathing exercises, relaxation exercises, and home-based physical activity. Progressive relaxation techniques have gained prominence in chronic-disease care due to benefits such as reductions in anxiety and stress (3).

Exercise training constitutes the cornerstone of pulmonary rehabilitation, enhancing physical performance, social integration, and independence. While short-term bronchodilation can be achieved with certain medical therapies, long-term lung function and exercise capacity typically continue to decline as the disease progresses. Consequently, structured physical exercise programmes are increasingly recognised as essential components of COPD management. Exercise training can stabilise clinical symptoms, slow disease progression, and is considered a mainstay of management for patients with moderate to severe COPD (stage II and above) (5).

According to the World Health Organization (WHO), approximately 65 million people have moderate to severe COPD, representing a substantial global burden. COPD affects an estimated 10%–20% of adults aged 40 years and older, contributing to more than 3 million deaths annually. Projections suggest that COPD will become the third leading cause of death worldwide by 2030 (6).

In Egypt, COPD prevalence is notably high, affecting around 7.5% of the population (7). The burden is greater in men than in women, and prevalence increases with age. Various factors contribute to the elevated prevalence in Egypt, including widespread smoking, air pollution, and occupational hazards (8).

Given the high COPD burden and constraints on healthcare resources in Egypt, there is a critical need for effective, low-cost, evidence-based rehabilitation strategies. This study aims to evaluate the effect of progressive relaxation exercises on dyspnea, anxiety, and peripheral oxygen saturation in COPD patients. The findings may offer valuable insights for promoting controlled breathing, reducing dyspnea and anxiety, and improving oxygen saturation, thereby providing a simple and cost-effective approach to enhancing quality of life.

## Materials and methods

### Study design

This study employed a quasi-experimental, single-group, pretest–posttest design. Participants were assessed prior to the intervention and subsequently reassessed after completion of the intervention to evaluate its effects.

### Study setting

The study was conducted at the chest department and outpatient clinic at Ain Shams University Medical Hospitals in Cairo, Egypt.

### Study period

The study was conducted over a period of six months, from December 2024 to May 2025. Recruitment and baseline assessments were completed during this period, with follow-up assessments conducted at one month and three months post-intervention to assess the durability of effects.

### Study population

The study included adult patients of all genders diagnosed with COPD and attending at the chest department and outpatient clinic at Ain Shams University Hospitals in Cairo, Egypt.

The inclusion criteria for this study required participants to be adult patients of either gender who had been recently diagnosed with mild or moderate COPD at the chest department and outpatient clinic in Ain Shams University Hospitals in Cairo, Egypt, who had not previously received progressive relaxation exercises and were eligible and available during the period from the beginning of December 2024 to May 2025. They were included in the study upon their acceptance to participate. However, patients with severe COPD or respiratory diseases that may limit ventilation during exercise performance (e.g., pulmonary resection, bronchiectasis, pulmonary hypertension, interstitial lung disease), patients with newly diagnosed tumours or those with major cognitive impairments like dementia, those not willing to participate, and/or those unpaid volunteers were excluded.

### Sample size and sampling technique

The target sample size was 60 participants. This was determined using a standard single-group sample size calculation based on the total number of COPD patients treated at Ain Shams University Hospitals in 2024 (*N* = 152), with a 95% confidence level (*z* = 1.96), an acceptable margin of error of 10% (*d* = 0.10), and an estimated population proportion of 0.50 (P). The 10% margin of error was selected in consideration of the exploratory, preliminary nature of the study and the feasibility constraints related to time and participant availability. A 60-participant sample provides the required precision for the primary pre–post analysis given the study's design. A purposive sampling technique was employed to recruit participants who met predefined inclusion and exclusion criteria and were available during December 2024 to May 2025. This approach allowed for the targeted recruitment of COPD patients suitable for the progressive relaxation exercises intervention. There were no dropouts in this study, as all participants completed the intervention until the end to follow up during the three-month study period. Adherence to the intervention was monitored through scheduled follow up visits and regular telephone contact to reinforce participation and confirm continued engagement with the progressive relaxation exercises. Attendance at assessment sessions (baseline, 1 month, and 3 months) was documented, and all enrolled participants completed the intervention and outcome assessments.

### Data collection

After obtaining approval from the general administration of Ain Shams University Hospitals in the Egyptian population and Ministry of Health, data were collected via face-to-face interviews conducted in the chest department and outpatient clinic. The questionnaire took approximately 20–25 minutes to complete by the participants (patients), not by nurses. Data were collected using four main instruments as follows:

#### Part I: A structured patient interview questionnaire

A structured patient interview questionnaire, developed by the investigators in English based on a review of related literature (9–11). This questionnaire gathered demographic data, including age, gender, marital status, educational level, occupation and nature of work, residence and living conditions, and income.

#### Part II: dyspnea-12 (D-12) questionnaire

A standardised instrument was adapted and available in English from Yorke et al. (12) and Arabic from Alyami et al. (13). It was used to evaluate the severity and qualitative aspects of dyspnea, including both physical and emotional components. The tool includes 12 items divided into a physical domain (items 1–7) and an emotional domain (items 8–12). Each item is scored on a four-point scale ranging from 0 (no dyspnea) to 3 (severe dyspnea). The total score ranges from 0 to 36, with scores of 1–12 indicating mild dyspnea, 13–24 indicating moderate dyspnea, and 25–36 indicating severe dyspnea.

#### Part III: beck anxiety inventory (BAI)

The Beck Anxiety Inventory (BAI) is standardised self-report instrument for measuring the severity of anxiety in adults. The English version was adapted from Bardhoshi et al. (14) and the Arabic version from Abdelsaid et al. (15) were employed. The BAI consists of 21 items rated on a 0–3 Likert scale, assessing emotional, cognitive, and physiological symptoms of anxiety. The total score ranges from 0 to 63, interpreted as mild anxiety (0–21), moderate anxiety (22–35), and severe anxiety (36–63).

#### Part IV: progressive relaxation exercises performance checklist

Finally, a performance checklist for progressive relaxation exercises, developed by the researchers in English based on related literature (16, 17). It was designed to evaluate the patient's ability to perform the steps of the exercises correctly. The checklist comprises 16 items; each item is scored as “done” or “not done”. The total score is 16 points, with one point awarded for each completed step. A score of 80% or more (13 points or higher) is considered a satisfactory level of performance, while a score below 80% indicates an unsatisfactory performance level.

### Progressive relaxation exercises course

Participants were approached during their visits to the chest department and outpatient clinic at Ain Shams University Hospitals, Cairo, Egypt. The study purpose and the progressive relaxation exercises (PREs) programme were explained in detail. All participants were assigned to a single group, and baseline measurements were collected using the pre-designed study tools after initial assessment. The progressive relaxation exercises programme was then tailored and initiated for each patient.

Researchers demonstrated the application in front of patients individually, so the researchers took two sessions. The first session started by explaining the objectives of the session and included the definition of progressive relaxation exercises (PREs), how to apply PREs, and the identified advantages & adverse effects and complications of PREs. It took 30 min for each patient. Then the second session included the patients re-demonstrating the procedure under the investigators' observation to ensure that they apply PREs correctly. It took 15–30 min for each patient.

Finally, each patient was asked to complete the course of PREs for one month 15–30 min application once a day. Otherwise, the investigator asked for the patient's telephone number to follow up with them and determined the second appointment after one month and after three months for follow-up in order to complete the data collection process.

#### Content validity and reliability

The validity of the study instruments was established through both face and content validity. Content validity aimed to examine whether each instrument accurately measured the intended concepts and adequately covered the study objectives. The questionnaire was determined by a panel of five experts in medical-surgical nursing, three professors from the department of medical-surgical nursing, Ain Shams University. One Faculty of Medicine, Ain Shams University and one clinical pulmonologist from the chest department Ain Shams University Hospital. The experts revised the instruments for clarity, relevance, understanding, and applicability; Based on their feedback, minor modifications were made to enhance the quality and accuracy of the instruments. Since the original instruments were developed in English, cultural and linguistic adaptation was performed to ensure appropriateness for the Egyptian healthcare context. In addition, the reliability of the instruments developed was tested using Cronbach's alpha coefficients to assess internal consistency for each instrument and for a predefined total instrument composite. The total alpha refers to the aggregated score combining the Dyspnea-12 (12 items), the Beck Anxiety Inventory (21 items), and the progressive relaxation exercises checklist (16 items) into a single composite outcome for the primary analysis. The total Cronbach's alpha was 0.822, indicating acceptable internal consistency for the composite. Individual alphas were 0.753 (D-12), 0.878 (BAI), and 0.858 (progressive relaxation checklist).

Additionally, the validity scores of the tools were 0.783 for the Dyspnea-12 questionnaire, 0.913 for the BAI, 0.852 for the progressive relaxation exercises performance checklist, and 0.835 for the total questionnaire. These values indicate a high level of internal consistency and strong validity across all study instruments.

#### Pilot study

A pilot study was carried out in December 2024, and was carried out in 10% of the sample size (6 patients with COPD) who participated in a pilot study to evaluate the applicability, clarity, and effectiveness of the tools and to estimate the time to fill it, which ranged between about 20–25 min. No data from the pilot were included in the main analyses, and no adjustments to the study instruments were made based on the pilot findings. The pilot sample was dedicated to pretesting only and did not contribute to the final findings.

## Ethics statement

This study was conducted in accordance with the ethical principles of the Declaration of Helsinki and its later amendments. A formal approval was obtained from the Research Ethic Committee of the Faculty of Nursing, Ain Shams University, Registered number 25.08.857, to carry out the study. Additionally, written informed consent was obtained before completing the questionnaire, COPD patients were informed that participation in the study is voluntary, and the confidentiality and anonymity of the information were confirmed.

### Data analysis

The SPSS software, version 27 was used for the statistical analysis. Characteristics of the sample were described by using descriptive statistics. Frequencies and percentages were used to describe different categorical variables, whereas means and standard deviations (SD) were used to represent continuous variables. The chi-square (*χ*2) test was used for analysis to compare proportions between qualitative parameters. A one-way analysis of variance (ANOVA) when comparing between more than two means. Independent-samples *t*-test of significance was used when comparing between two means. Pearson's correlation coefficient (*r*) test was used to assess the degree of association between two sets of variables. *p*-value <0.05 was considered significant, *p*-value <0.001 was considered as highly significant, *p*-value >0.05 was considered insignificant.

## Results

A total of 60 patients with chronic obstructive pulmonary disease (COPD) were enrolled in the study, including participants from the pilot phase, with no subsequent changes to the study protocol. Table 1 shows that 50% of the studied patients were aged between 35 and 50 years, with a mean age of 46.05 ± 8.68 years and a range of 20–64 years; 88.3% were male, and 75% were married. Regarding educational attainment, 51.7% had completed secondary education. In terms of employment, 71.7% were employed, and 76.7% were engaged in occupations involving exposure to respiratory irritants. 55% resided in urban areas, and 56.7% lived near a source of pollution, with 70.6% of them identifying the source as being located in crafts or industrial areas. 71.7% of the studied patients were current smokers, and among them, 72.1% reported a smoking duration of 10 years or more.

**Table 1:** Socio-demographic characteristics of the study participants.

Variables	N (%)
**Age “years”**	
2035 years	11 (18.3)
>35–50 years	30 (50.0)
>50–64 years	19 (31.7)
Mean ± SD	46.05 ± 8.68
**Gender**	
Male	53 (88.3)
Female	7 (11.7)
**Marital status**	
Single	15 (25)
Married	45 (75)
**Level of education:**	
Reads and writes	15 (25.0)
Secondary school	31 (51.7)
Higher education	14 (23.3)
**Work status:**	
Employed	43 (71.7)
Unemployed	17 (28.3)
If employed, what is the nature of work? (*n* = 43)	
Work that exposes to respiratory irritants	33 (76.7)
Office work	10 (23.3)
**Residence:**	
Rural	27 (45.0)
Urban	33 (55.0)
**Do you live near a source of pollution?**	
Yes	34 (56.7)
No	26 (43.3)
**If yes, what is the source of pollution? (*n* = 34)**	
Factories	10 (29.4)
Crafts/ industrial area	24 (70.6)
**Do you smoke**	
Yes	43 (71.7)
No	17 (28.3)
**If yes since, when did you start smoking (*n* = 43)**	
Less than 5 years	1 (2.3)
From 5 years to less than 10 years	11 (25.6)
10 years and more	31 (72.1)

Data are expressed as means ± SD for continuous variables and as percentages for different categorical variables. SD, Standard Deviation; COPD, Chronic obstructive pulmonary disease.

Table 2 shows that 53.3% of the studied patients had been diagnosed with COPD for 1 to 5 years. Among the most commonly reported symptoms at the time of diagnosis, 90% had difficulty breathing, 100% were undergoing investigation for the illness, and 56.7% were admitted to hospital within 7–14 days.

**Table 2:** Distribution of study participants by COPD diagnosis duration and presenting symptoms.

Variables	N (%)
**Diagnosed with COPD**	
Less than one year	19 (31.7)
From 1 to 5 years	32 (53.3)
More than 5 years	9 (15.0)
**Signs and symptoms when you are diagnosed with COPD**	
Difficulty in breathing	54 (90.0)
Tachypnea	21 (35.0)
Increased sputum production	36 (60.0)
Chronic cough	42 (70.0)
**Duration of hospital admission**	
3–7 days	21 (35.0)
>7–14 days	34 (56.7)
>14 days	5 (8.3)

Data are expressed as average scores for continuous variables and as (%) percentages for different categorical variables. N, Number; COPD, Chronic obstructive pulmonary disease.

**Figure 1:**
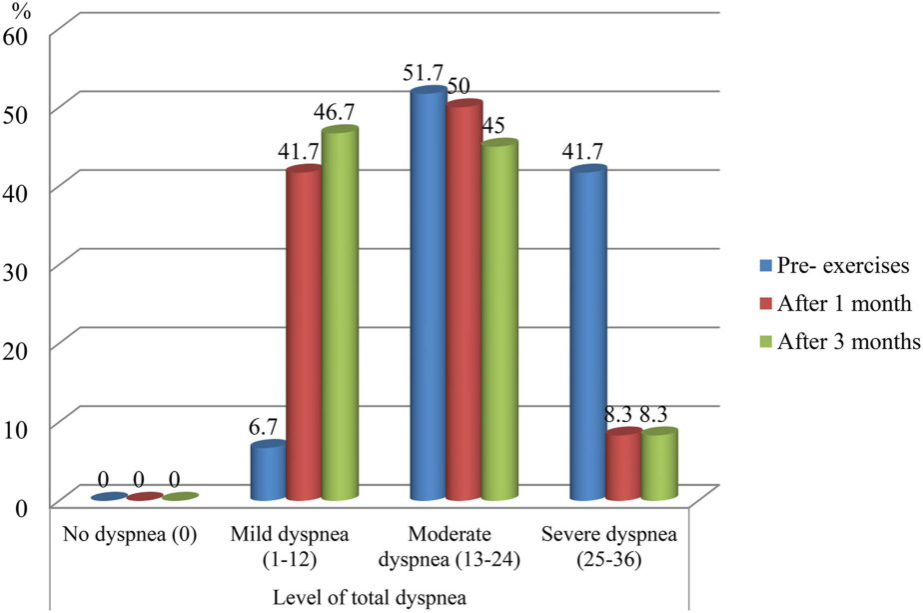
Percentage of studied COPD patients (pre-exercises and post-exercises) according to their level on the dyspnea 12 (D-12) questionnaire.

Figure 1 shows the distribution of dyspnea levels among studied COPD patients at different time points. At baseline (pre-exercise), 41.7% reported severe dyspnea. After one month of exercise, 50.0% reported moderate dyspnea, and after three months, 46.7% reported mild dyspnea (Figure 1).

**Figure 2:**
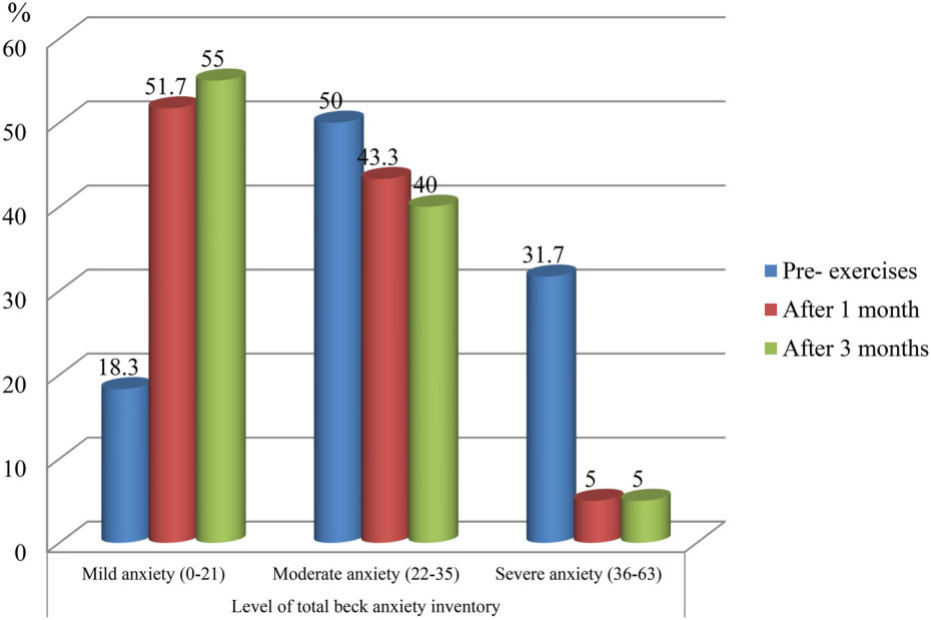
Percentage of studied COPD patients (pre-exercises and post-exercises) according to their total BAI anxiety level.

Figure 2 illustrates the distribution of anxiety levels among studied COPD patients at baseline and after subsequent exercise periods. At baseline, 50.0% reported moderate anxiety before the exercises. After one month, there was a marked shift, with mild anxiety increasing to 51.7%. This trend continued after three months, with mild anxiety rising further to 55.0% (Figure 2).

**Figure 3:**
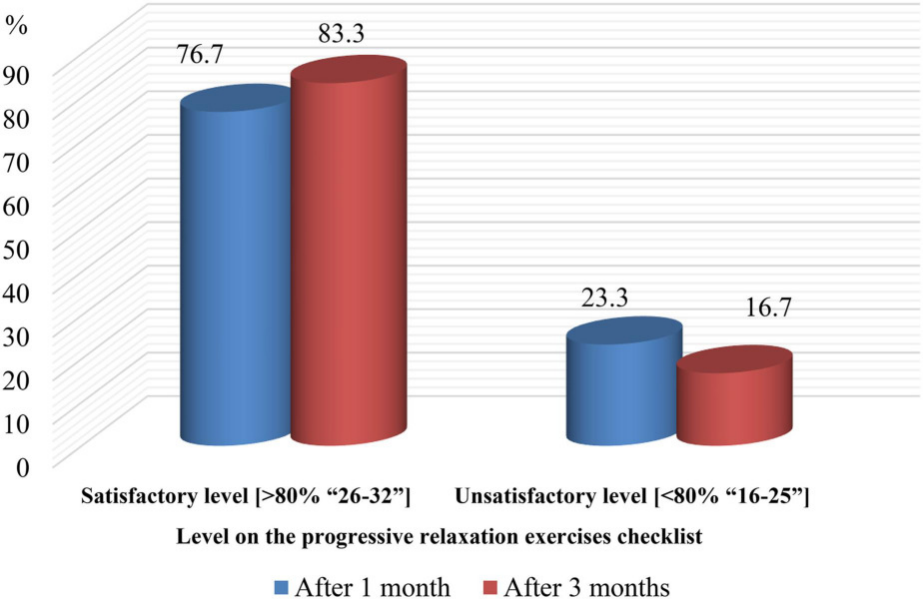
Percentage of studied COPD patients (pre-exercises and post-exercises) according to their level on the progressive relaxation checklist.

Figure 3 shows the distribution of performance on the progressive relaxation checklist among studied COPD patients. After one month of exercise, 76.7% demonstrated a satisfactory level of performance, increasing to 83.3% at the three-month follow-up (Figure 3).

Table 3 shows significant associations between the total Dyspnea-12 score, the total Beck Anxiety Inventory (BAI) score, the Progressive Relaxation Exercise (PRE) checklist score, and oxygen saturation (SaO2) after one month. The signs of the correlations indicate that higher Dyspnea-12 and higher BAI scores are associated with lower SaO2, whereas higher PRE checklist scores are associated with higher SaO2. In other words, some pairwise relationships are negative and others are positive.

**Table 3:** Correlation between total score of dyspnea 12 questionnaire, BAI, and performance checklist for progressive relaxation exercises after 1 month (*N* = 60).

After 1 months - (*N* = 60)					
Variable test of significance		Total mean score of D-12 questionnaire	Total mean score of BAI	Total means score of the performance checklist for PRE	SaO2 level
Total mean score of the D-12 questionnaire	*r*-value		0.469	0.515	−0.417
	*p*-value		0.003*	<0.001**	<0.001**
	*N*		60	60	60
Total means score of the BAI	*r*-value	0.469		0.485	−0.684
	*p*-value	0.003*		0.002*	<0.001**
	*N*	60		60	60
Total mean score of the performance checklist for PRE	*r*-value	0.515	0.485		0.527
	*p*-value	<0.001**	0.002*		<0.001**
	*N*	60	60		60
SaO2 level	*r*-value	−0.417	−0.684	0.627	
	*p*-value	<0.001**	<0.001**	<0.001**	
	*N*	60	60	60	

*r*-Pearson Correlation Coefficient; The chi-square (*χ*2) test was used to examine differences in the prevalence of different categorical variables.

* *p*-value <0.05 significant correlation;.

** *p*-value <0.001 highly significant.

D-12, Dyspnea 12; BAI, Beck Anxiety Inventory; PRE, progressive relaxation exercises; SaO2, Oxygen Saturation.

Table 4 indicates that there was a statistically negative correlation between total score of the Dyspnea 12 questionnaire, the total mean score of the BAI, the performance checklist for progressive relaxation exercises and the oxygen saturation level after 3 months.

**Table 4:** Correlation between total score of dyspnea 12 questionnaire, beck anxiety inventory, and performance checklist for progressive relaxation exercises after 3 months (*N* = 60).

After 1 months - (*N* = 60)					
Variable test of significance		Total mean score of D-12 questionnaire	Total mean score of BAI	Total means score of the performance checklist for PRE	SaO2 level
Total mean score of the D-12 questionnaire	*r*-value		0.422	0.505	−0.448
	*p*-value		0.004*	<0.001**	<0.001**
	*N*		60	60	60
Total means score of the BAI	*r*-value	0.422		0.437	−0.736
	*p*-value	0.004*		0.003*	<0.001**
	*N*	60		60	60
Total mean score of the performance checklist for PRE	*r*-value	0.505	0.437		0.674
	*p*-value	<0.001**	0.003*		<0.001**
	*N*	60	60		60
SaO2 level	*r*-value	−0.448	−0.736	0.674	
	*p*-value	<0.001**	<0.001**	<0.001**	
	*N*	60	60	60	

r-Pearson Correlation Coefficient; The chi-square (*χ*2) test was used to examine differences in the prevalence of different categorical variables.

* *p*-value <0.05 significant correlation;.

** *p*-value <0.001 highly significant.

D-12, Dyspnea 12; BAI, Beck Anxiety Inventory; PRE, progressive relaxation exercises; SaO2, Oxygen Saturation.

## Discussion

Dyspnea, anxiety, and oxygen saturation are critical clinical targets in COPD management, given their impact on function and quality of life. Dyspnea remains one of the most disabling COPD symptoms, often limiting activity and contributing to reduced quality of life. Anxiety commonly accompanies breathlessness, potentially creating a self-perpetuating cycle that worsens both physical and psychological well-being (18).

In this study, we employed a quasi-experimental design (pretest and posttest intervention) to evaluate the effect of progressive relaxation exercises (PREs) on dyspnea, anxiety, and oxygen saturation among patients with COPD. Participants underwent baseline assessments, followed by participation in a structured PREs program, and subsequent assessments to determine the intervention's impact. The approach allowed us to examine whether implementing PREs can alleviate dyspnea and anxiety while stabilizing or improving peripheral oxygen saturation in this population.

Patients' dyspnea pre-, post-, and follow-up exercises. The current study indicated that before the exercises, more than half of the studied patients experienced moderate dyspnea, or more than two-fifths experienced severe dyspnea. One month after the intervention, there was a marked reduction in severe dyspnea, with this level becoming negligible, and the improvement was sustained at the three-month follow-up. The studied patients experiencing mild dyspnea increased substantially from the few patients to nearly two-fifths after one month and improved to less than half in follow-up after three months.

These findings are consistent with the beneficial effects of structured exercise training on respiratory muscle function, ventilation efficiency, and the perceptual burden of breathlessness. Regular practice of progressive relaxation and controlled breathing may also attenuate anxiety, enhance oxygen exchange, and improve overall physical tolerance, collectively contributing to the observed reduction in severe dyspnea and a shift toward milder symptoms over time.

These findings are consistent with Zare et al. (19) who found that breathing exercises improved respiratory function in COPD patients. Similarly, Abdi Alvar et al. (20) reported that breathing exercises improved pulmonary indices.

Regarding anxiety as measured by the Beck Anxiety Inventory (BAI), our study found that before the exercises, about half of the studied patients experienced moderate anxiety, with nearly one-third experiencing severe anxiety and a minority reporting mild anxiety. One month after the intervention, there was a marked shift: mild anxiety increased to more than half of participants, and severe anxiety decreased to only a few patients. At the three-month follow-up, mild anxiety rose further to approach two-thirds, while severe anxiety remained low in a few patients.

Regular practice of progressive relaxation and controlled breathing likely contributed to reductions in anxiety, improved oxygen exchange, and enhanced physical tolerance, collectively supporting the observed improvements in dyspnea and shifts toward milder symptoms over time.

The observed improvement can be attributed to the beneficial effects of relaxation and breathing exercises, which help reduce physiological stress responses, promote calmness, and enhance coping with respiratory difficulties. The exercises likely reduced the fear and psychological distress associated with breathlessness, improved patients' sense of control over their condition, and strengthened overall emotional well-being. Sustained practice over time appears to have reinforced these benefits, contributing to a continued reduction in severe anxiety and a progressive shift toward milder anxiety levels.

These findings are consistent with Liu & Xu (21). who reported that therapeutic controlled breathing exercises (TCE) significantly improve lung function and reduce anxiety and depression in COPD patients. They also align with Volpato et al. (22) who observed that moderate and severe anxiety were more prevalent in the study group compared with control groups, whereas the absence of anxiety was higher in the negative control group than in the other groups, and mild anxiety was more common in the negative control group relative to the positive control and study groups.

Progressive Relaxation Exercises Performance Checklist. The current study found that more than three-quarters of participants demonstrated a satisfactory level of performance on the progressive relaxation exercises (PREs) checklist, with the proportion increasing to a majority by the three-month follow-up. This sustained improvement suggests that the intervention not only produced immediate gains in performing PREs but also promoted continued adherence and skill retention over time. The observed enhancement in performance may reflect improved physical capacity, greater respiratory efficiency, and strengthened self-management skills, leading to better symptom control, reduced anxiety, and increased confidence in performing daily activities. The durability of these gains at follow-up indicates that participants applied and maintained the learned techniques beyond the supervised sessions, contributing to longer-term functional benefits and overall well-being.

These findings are broadly in line with Sadek et al. (23) who reported a shift from low to high levels of practice in deep-breathing techniques and related self-management behaviours following a structured programme. Specifically, Sadek and colleagues observed that while a minority initially demonstrated satisfactory practice of certain techniques (e.g., deep breathing and coughing), most participants achieved satisfactory practice post-intervention and at follow-up, similar to the pattern seen in our PREs program.

The correlations observed among dyspnea, anxiety (BAI), and progressive relaxation performance were positive at both one- and three-month time points, indicating that greater symptom burden tended to coincide with higher anxiety and with higher engagement or performance on the relaxation checklist. This pattern could reflect several scenarios: individuals with higher dyspnea and anxiety may have been more adherent to or benefited more from the relaxation programme, or there may be concurrent increases in both symptoms reporting and engagement with the exercises. The association with oxygen saturation differed over time: at one-month, higher dyspnea, higher anxiety, and higher performance scores aligned with higher oxygen saturation; by three months, this pattern reversed, with higher dyspnea, higher anxiety, and higher performance scores co-occurring with lower oxygen saturation. These time-dependent shifts suggest that the relationships among dyspnea, anxiety, exercise performance, and oxygenation evolve during the intervention and should be interpreted in light of measurement timing, potential response shifts, and confounding factors.

The observed improvements reinforce the beneficial impact of progressive relaxation exercises over time, with better performance on the relaxation tasks associated with lower dyspnea and lower anxiety scores, reinforcing the role of PREs as a complementary approach in COPD management. These findings align with those of Neşe & Bağlama (3), who reported statistically significant positive correlations before and after PRE and deep breathing exercise intervention applications.

Also, these findings agreed with Shiraishi et al. (24) reported a significant positive correlation between functional status and the level of dyspnea, suggesting that better function is associated with lower perceived breathlessness. Likewise, similar findings were reported in a study conducted by Rutkowski et al. (25) documented that range-of-motion exercises constitute a meaningful standard treatment for COPD, reducing symptoms, increasing walking capacity, and improving quality of life. In line with this context, Paolucci et al. (26) demonstrated that a four-week supervised, intensive pulmonary rehabilitation programme including endurance and strength training- improved dyspnea, exercise excursion, and quality of life (QoL). Likewise, Yang (27), highlighted that physical inactivity is a significant problem in COPD, with reductions in physical activity beginning early in the disease trajectory. Collectively, these studies support the notion that structured rehabilitation and maintained physical activity contribute to better symptom control and overall outcomes in COPD.

### Strengths and limitations

The study indicates a meaningful pattern of improvement across dyspnea, anxiety, and functional performance following progressive relaxation among COPD patients, with notable gains remaining at three months and a strong connection between anxiety, age, and performance. It adds useful preliminary evidence on the feasibility and potential effectiveness of introducing relaxation techniques into COPD management, backed by repeated measures at several time periods. However, the study is not without limitations. Our current study was carried out at a single recruitment location, which may not adequately represent the comprehensive population of patients with COPD, which may be affecting generalizability. The capacity to fully ascribe reported interference is limited by the quasi-experimental, single-group pretest–posttest design without a control group since other concurrent factors may have influenced outcomes. Without a comparison group, it is not possible to determine whether the observed improvements were attributable to the progressive relaxation intervention itself or to other concurrent factors, such as natural disease fluctuation, regression to the mean, increased clinical attention, medication adjustments, or external lifestyle changes. The absence of randomization further increases susceptibility to confounding, and both measured and unmeasured variables may have influenced the outcomes. Consequently, the findings should be interpreted as associative rather than causal. Finally, the three-month follow-up period may be insufficient to determine the long-term sustainability of the observed benefits.

## Conclusion

In our study, it can be concluded that more than half of the studied patients experienced moderate dyspnea pre-exercise, which improved to mild dyspnea post-exercise and in follow-up after three months. Nearly half experienced moderate anxiety pre-exercise, which improved to mild anxiety post-exercise, and after three months, severe anxiety remained low in a few patients. More than three-quarters of the studied patients demonstrated a satisfactory level of performance, which further increased to the majority at the three-month follow-up. There was a statistically significant relation between anxiety scores and age groups before the exercises.

Furthermore, there was a statistically significant relation between the studied patients with COPD at the pre-intervention, one-month, and three-month intervals, their performance, and socio-demographic data. There was a statistically significant positive correlation between the total scores of dyspnea, the BAI, and the performance checklist after one month of implementing progressive relaxation exercises among patients with COPD.

## Data Availability

The raw data supporting the conclusions of this article will be made available by the authors, without undue reservation.

## References

[1] Shahpasand M Mohammadpour A Najafi S Sobhani M. Effect of local hyperthermia on respiratory indices of patients with chronic obstructive pulmonary disease. Iran J Nurs Midwifery Res. (2023) 28(1):110–7. 10.4103/ijnmr.ijnmr_381_20 37250949 PMC10215542

[2] Mu H Zhang Q. The application of diaphragm ultrasound in chronic obstructive pulmonary disease: a narrative review. COPD J Chronic Obstr Pulm Dis. (2024) 21(1):2331202. 10.1080/15412555.2024.2331202 38634575

[3] Neşe A Samancıoğlu Bağlama S. The effect of progressive muscle relaxation and deep breathing exercises on dyspnea and fatigue symptoms of COPD patients: a randomized controlled study. Holist Nurs Pract. (2022) 36(4):E18–26. 10.1097/HNP.0000000000000531 35708562

[4] Støve MP Graversen AH Sørensen J. Assessment of noninvasive oxygen saturation in patients with COPD during pulmonary rehabilitation: smartwatch versus pulse oximeter. Respir Care. (2023) 68(8):1041–8. 10.4187/respcare.10760 37193599 PMC10353168

[5] Wen Y Liu L Xun Z Jiaying M Shuyi C Wanwen H Effect of a rehabilitation garden on rehabilitation efficacy in elderly patients with chronic obstructive pulmonary disease. Pak J Zool. (2020) 52(6):2393. 10.17582/journal.pjz/20191204121245

[6] Peate I Hill B. Fundamentals of Critical Care: A Textbook for Nursing and Healthcare Students. New York: John Wiley & Sons (2022).

[7] Ali NR Omar Mohamed A. Risk factors of acute exacerbation of chronic obstructive pulmonary disease. Assiut Sci Nurs J. (2024) 12(47):101–10. 10.21608/asnj.2024.309139.1879

[8] Atef Mohamed N Abd Elkader Ahmed M Awadeen L. Assessment quality of life for patients with chronic obstructive pulmonary disease in outpatients clinic at Beni-Suef university hospital. Egypt J Health Care. (2023) 14(4):554–77. 10.21608/ejhc.2023.329537

[9] Assaf EA Badarneh A Saifan A Al-Yateem N. Chronic obstructive pulmonary disease patients’ quality of life and its related factors: a cross-sectional study of the Jordanian population. F1000Res. (2022) 11:581. 10.12688/f1000research.121783.1 35811805 PMC9237555

[10] Tyerman J Cobbett S Harding MM Kwong J Roberts D Hagler D Lewis’s medical-surgical nursing in Canada-E-Book: Lewis’s medical-surgical nursing in Canada-E-Book: Elsevier Health Sciences (2022).

[11] Stromberg H Dallred C Dewit S. Medical-Surgical Nursing E-Book: Concepts & Practice. Amsterdam: Elsevier Health Sciences (2021).

[12] Yorke J Swigris J Russell A-M Moosavi SH Kwong GNM Longshaw M Dyspnea-12 is a valid and reliable measure of breathlessness in patients with interstitial lung disease. Chest. (2011) 139(1):159–64. 10.1378/chest.10-0693 20595454 PMC3035488

[13] Alyami MM Jenkins SC Lababidi H Hill K. Reliability and validity of an arabic version of the dyspnea-12 questionnaire for Saudi nationals with chronic obstructive pulmonary disease. Ann Thorac Med. (2015) 10(2):112–7. 10.4103/1817-1737.150730 25829962 PMC4375739

[14] Bardhoshi G Duncan K Erford BT. Psychometric meta-analysis of the English version of the beck anxiety inventory. J Couns Dev. (2016) 94(3):356–73. 10.1002/jcad.12090

[15] Abdelsaid JY El-Geneidy MM Maximos MH Abd El-Salam RM. Effect of progressive muscle relaxation technique on blood pressure, anxiety and stress among elders in assisted living facilities. Alex Sci Nurs J. (2019) 21(2):69–82. 10.21608/asalexu.2019.206598

[16] Norelli S Long A Krepps J. Relaxation techniques. In: StatPearls. Treasure Island (FL): StatPearls Publishing (2024). 30020610

[17] Toussaint L Nguyen QA Roettger C Dixon K Offenbächer M Kohls N Effectiveness of progressive muscle relaxation, deep breathing, and guided imagery in promoting psychological and physiological states of relaxation. Evid Based Complement Alternat Med. (2021) 2021(1):5924040. 10.1155/2021/5924040 34306146 PMC8272667

[18] Qiu C-J Wu S. Depression and anxiety disorders in chronic obstructive pulmonary disease patients: prevalence, disease impact, treatment. World J Psychiatry. (2024) 14(12):1797. 10.5498/wjp.v14.i12.1797 39704377 PMC11622031

[19] Zare F Karimyar Jahromi M Rahmanian Z Faseleh Jahromi M. Comparing effects of breathing exercises alone and combined with breathing-stretching exercises on respiratory indices, disease severity and exercise capacity in COPD. Sci Rep. (2025) 15(1):5068. 10.1038/s41598-025-89664-z 39934204 PMC11814402

[20] Abdi AD Kalroozi F Nezamzadeh M Pishgooie S. The effect of controlled breathing exercises on anxiety and arterial oxygen saturation in chronic obstructive pulmonary disease the military specialist hospitals. Mil Caring Sci. (2020) 7(2):96–105. 10.29252/MCS.7.2.96

[21] Liu S Xu D. Effects of traditional Chinese exercise on lung function and mental health in patients with COPD: a systematic review and meta-analysis. Front Public Health. (2025) 13:1612741. 10.3389/fpubh.2025.1612741 40709047 PMC12286970

[22] Volpato E Toniolo S Pagnini F Banfi P. The relationship between anxiety, depression and treatment adherence in chronic obstructive pulmonary disease: a systematic review. Int J Chron Obstruct Pulmon Dis. (2021) 16:2001–21. 10.2147/COPD.S313841 34262270 PMC8275112

[23] Sadek Ramadan S Nagah Hasan Mohamed S. Effect of nurse-led pulmonary rehabilitation program on dyspnea and fatigue for patients with chronic obstructive pulmonary disease. Egypt J Health Care. (2021) 12(2):608–29. 10.21608/EJHC.2021.167096

[24] Shiraishi M Higashimoto Y Sugiya R Mizusawa H Takeda Y Fujita S Diaphragmatic excursion correlates with exercise capacity and dynamic hyperinflation in COPD patients. ERJ Open Res. (2020) 6(4):00589-2020. 10.1183/23120541.00589-2020 33447614 PMC7792831

[25] Rutkowski S Rutkowska A Kiper P Jastrzebski D Racheniuk H Turolla A Virtual reality rehabilitation in patients with chronic obstructive pulmonary disease: a randomized controlled trial. Int J Chron Obstruct Pulmon Dis. (2020) 15:117–24. 10.2147/COPD.S223592 32021150 PMC6968810

[26] Paolucci T Patrizio G Pietrantonio D Rapacchiale G Spacone A Parruti G Utility of high flow nasal cannula during pulmonary rehabilitation in COVID-19 patients in acute respiratory failure. Appl Sci. (2022) 12(9):4637. 10.3390/app12094637

[27] Yang T. Expression profile of IL-17 in lung tissues of patients with lung cancer and COPD and clinical significance. Cell Mol Biol. (2023) 68(9):135–9. 10.14715/cmb/2022.68.9.21 36905261

